# Parastomal hernia repair using retromuscular 3D funnel mesh: the “Sugar Funnel” technique

**DOI:** 10.1007/s10029-025-03543-0

**Published:** 2026-01-09

**Authors:** Alaa Soliman, Gaurav V. Kulkarni, David Barnes, Toby M. Hammond

**Affiliations:** 1 Department of General and Colorectal Surgery, Broomfield Hospital, Mid and South Essex NHS Foundation Trust, Court Road, Chelmsford, Essex UK; 2 York Abdominal Wall Unit, Department of General Surgery, York Teaching Hospital NHS Foundation Trust, Wigginton Road, York, YO31 8HE UK; 3 Department of Plastic Surgery and Burns (St Andrew’s Centre), Broomfield Hospital, Mid and South Essex NHS Foundation Trust, Court Road, Chelmsford, Essex UK

**Keywords:** Parastomal hernia, Retromuscular repair, Transversus abdominis release, 3D funnel mesh, Abdominal wall reconstruction, Abdominoplasty

## Abstract

**Introduction:**

Parastomal hernias are a common and complex problem for patients and their surgeons. Despite multiple established repair strategies, recurrence rates remain high. We present a novel open retromuscular repair technique using a 3D funnel mesh.

**Methods:**

The technique involves retrorectus dissection, transversus abdominis release (TAR), lateralisation of the stomal conduit, and retromuscular placement of a 3D funnel mesh. A second flat mesh reinforces the remaining retromuscular space. Abdominoplasty was performed where excess skin or fat contributed to the parastomal ‘bulge’.

**Results:**

Fifteen patients underwent repair (median age 59 years, BMI 33.8 kg/m²). Nine had a colostomy and six an ileostomy. Five (33%) had a recurrent parastomal hernia, and 13 (87%) had concomitant incisional hernia. The median combined transverse defect width was 11.9 cm (IQR 10.4–12.2). Abdominoplasty was performed in 13 patients (87%). At median 15-month follow-up, no recurrences, stenoses, or obstructions were observed. Complications included wound infection (*n* = 2), seroma (*n* = 1), abscess (*n* = 1), stoma prolapse (*n* = 1), and stoma retraction (*n* = 1). Two patients required stoma refashioning.

**Conclusion:**

The retromuscular 3D funnel mesh technique offers a robust repair with potential advantages over other recognized repair options. It also addresses the peristomal ‘bulge’ of redundant skin and excess subcutaneous fat that can compromise stoma appliance application and adherence. Comparative studies focused on patient reported outcome measures are needed to determine if this technique confers advantages over other established procedures.

## Introduction

Parastomal hernia (PSH) repair is technically demanding. It must fulfil the principles of incisional hernia repair, which include reduction of hernia contents, excision of the hernia sac (unless needed as part of the repair) and mesh reinforced closure of the fascial defect, while preserving stoma function. The fascial trephine must be narrowed and mesh reinforced sufficiently to prevent recurrence yet left wide enough to avoid stomal obstruction or ischaemia. However, as the fascial trephine cannot be completely closed, some degree of recurrence after PSH repair may be regarded as inevitable.

There is broad consensus that the intraperitoneal and retromuscular planes are the preferred locations for mesh placement for PSH repair [1]. To address the specific complexities of PSH repair different mesh configurations have been described.

For intraperitoneal repair, which is usually performed by a minimally invasive approach, the options for mesh configuration include keyhole, modified Sugarbaker, the ‘Sandwich’ combination, and the 3D funnel mesh [2]. A recent systematic review and nationwide registry data have shown that the ‘Sandwich’ and 3D funnel mesh configurations achieve the lowest recurrence rates, with 3D funnel mesh associated with the lower rates of surgical-site occurrence (SSO) [2, 3].

Retromuscular repair, performed via open or minimally invasive approaches, is gaining favour owing to the avoidance of potential mesh-related visceral complications associated with the intraperitoneal approach. Techniques include retromuscular keyhole and Sugarbaker mesh configurations [4, 5]. A recent randomised controlled trial reported 2-year recurrence rates of 24% and 17%, respectively, with no significant differences in surgical site infection or reoperation [6]. The authors concluded that further innovation was required.

In this article we propose an open retromuscular repair using a permanent synthetic 3D funnel mesh. The approach aims to optimise both functional and reconstructive outcomes. The key operative steps are described, and our experience of the technique is reported.

## Methods

### Patient selection

The open retromuscular 3D funnel mesh repair was performed in carefully selected patients:

- Non-smokers, without active Crohn’s disease, and preferably with a body mass index (BMI) < 35.
- Patients with symptomatic parastomal hernia significantly impairing quality of life, in whom conservative measures had failed or were unlikely to be effective.
- Patients with either:
  - a parastomal fascial defect >5 cm, with or without a concomitant midline incisional hernia (EHS classification III or IV) [7], or.
  - a parastomal fascial defect ≤ 5 cm with a concomitant incisional hernia ≥ 4 cm (EHS PSH classification II and EHS incisional midline hernia classification W2 or W3) [7, 8].

All patients provided fully informed consent for surgery.

### Perioperative management

Patients with combined PSH and midline fascial defect widths > 8 cm received botulinum toxin A injections to the lateral abdominal wall muscles (300 IU, 150 IU per side) approximately 4 weeks before surgery to facilitate midline repair and reduce abdominal wall tension during reconstruction.

At the time of surgery, all patients received perioperative antibiotics according to hospital policy, venous thromboembolism prophylaxis (sequential compression devices and subcutaneous low molecular weight heparin), and intravenous tranexamic acid at induction and every 4 h thereafter depending on operative duration. Patients were placed supine on a warming mattress and covered with a warming device. A surgical-site infection prevention protocol was strictly followed.

Surgery was usually performed jointly by a general and colorectal surgeon and a plastic surgeon, both experienced in complex abdominal wall reconstruction.

### Surgical technique

The procedure involves retrorectus dissection, posterior component separation via transversus abdominis release (TAR), and retromuscular placement of a permanent synthetic 3D funnel mesh.

If a patient has excess peristomal skin and subcutaneous fat as part of the parastomal ‘bulge’, an abdominoplasty with or without umbilicoplasty, according to the patient’s preference, is planned. This may be a vertical, transverse or fleur de lis approach depending on the individual patient.

**Fig. 1:**
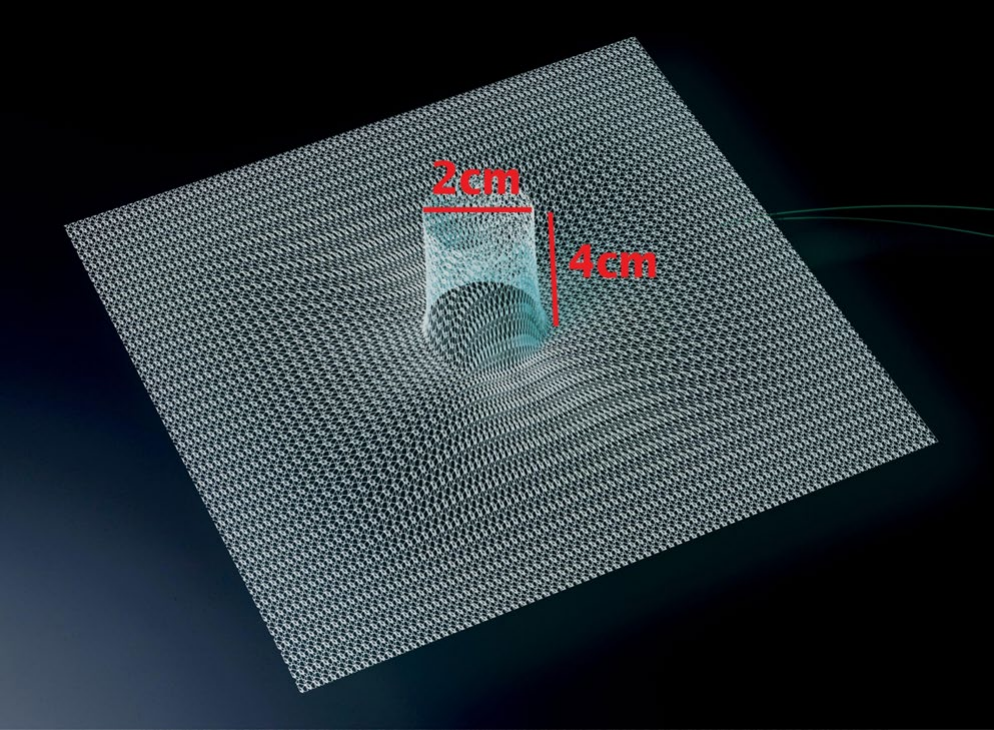
DynaMesh-IPST^®^ 3D funnel mesh used in the Sugar Funnel technique. The mesh consists of a flat reticular body (15 × 15 cm) with an integrated cylindrical funnel (4 cm in length, 2 cm in diameter) *Reproduced with permission from DynaMesh*^®^, *FEG Textiltechnik mbH*,* Aachen*,* Germany*

The 3D funnel mesh used was DynaMesh-IPST^®^, specifically the 15 × 15 cm size with a central funnel 2 cm in diameter and 4 cm in length (FEG Textiltechnik, Aachen, Germany) (Fig. 1). It is a permanent synthetic mesh composed of polyvinylidene fluoride and is specifically designed to prevent and repair parastomal hernias. It has a dual-layer composite structure and is only licensed for intraperitoneal placement. It is not licensed for placement in the retromuscular space, and the technique described is an ‘off-label’ use of the mesh.

The operation is divided into 5 stages:

Stage 1 – Stoma Release and Closure.

The peristomal skin is prepared with 2% chlorhexidine in 70% alcohol, and the area is draped. The stoma conduit is mobilised from the surrounding skin, subcutaneous tissue, and hernia sac contents to allow stapled closure using a transverse linear cutting stapler. An aqueous betadine-soaked swab is placed in the trephine over the stapled off stomal conduit.

Stage 2 – Laparotomy and Retromuscular Dissection.

The abdomen is re-prepped with 2% chlorhexidine in 70% alcohol, and new sterile drapes, gowns, gloves, and instruments are used. Depending on the operative plan, a vertical, transverse, or fleur-de-lis incision is performed.

The stapled stoma conduit and associated hernia sac contents are released from the subcutaneous tissue and fascial edges of the abdominal wall trephine and, when possible, reduced into the abdominal cavity. The parastomal and, if present, midline incisional hernia sacs are preserved in case they are required later.

A midline laparotomy is then performed with careful lysis of any adhesions to the anterolateral abdominal wall. If the patient has symptoms of intermittent obstruction, all small bowel adhesions are divided. If the stomal conduit and parastomal hernia contents could not be fully reduced previously this is completed. A large saline-soaked and wrung out swab is placed within the peritoneal cavity, spanning both paracolic gutters and extending from the pelvis to over the stomach and liver, to protect the viscera.

**Fig. 2:**
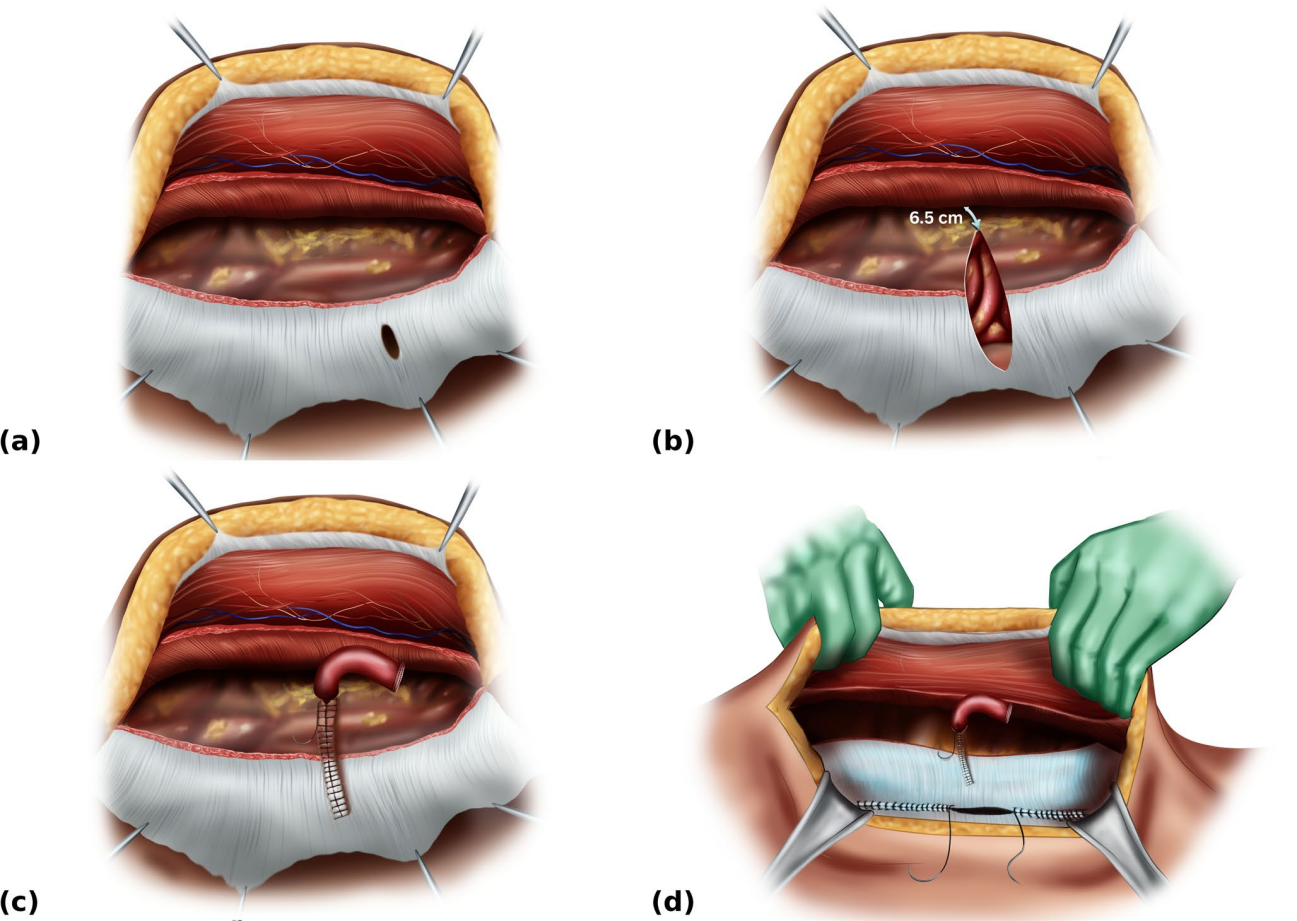
Retrorectus dissection with transversus abdominis release (TAR), posterior-layer closure, and stoma lateralisation. (**a**) Unilateral TAR on the stoma side showing the opening in the posterior layer through which the stomal conduit previously passed (**b**) Lateralisation of the opening in the posterior layer to a point 6.5 cm from the most lateral TAR dissection. This allows for placement of the 3D funnel mesh as it matches the distance from the edge of the central funnel to the outer edge of its flat portion (**c**) Closure of the lateralising incision in the posterior layer with a running suture, leaving a 2 cm lateral opening through which the stapled stomal conduit is drawn into the retromuscular space (**d**) Closure of the posterior layer in the midline from cranial and caudal directions, leaving an approximate 10 cm central gap

The retrorectus plane is developed, preserving perforating neurovascular bundles to the rectus abdominis. Posterior component separation is achieved via transversus abdominis release (TAR) on the side of the stoma (Fig. 2a), and contralaterally if required to facilitate midline closure.

Stage 3 – Stoma Lateralisation and Posterior Layer Closure.

The opening in the posterior layer (posterior rectus sheath, peritoneum, and transversalis fascia) through which the stomal conduit previously passed is lateralised. This is achieved by a linear incision carried laterally to a point 6.5 cm from the most lateral edge of the TAR dissection (Fig. 2b). This allows for placement of the 3D mesh as it matches the distance from the edge of the central funnel to the outer edge of its flat portion.

The lateralising incision in the posterior layer is closed with a running suture from medial to lateral, leaving a 2 cm lateral opening. The stapled stomal conduit is pulled through the lateral opening to sit in the retromuscular space (Fig. 2c),

The posterior layer is then closed in the midline from cranial and caudal directions, leaving an approximate 10 cm central gap (Fig. 2d).

Stage 4 – Mesh Placement.

**Fig. 3:**
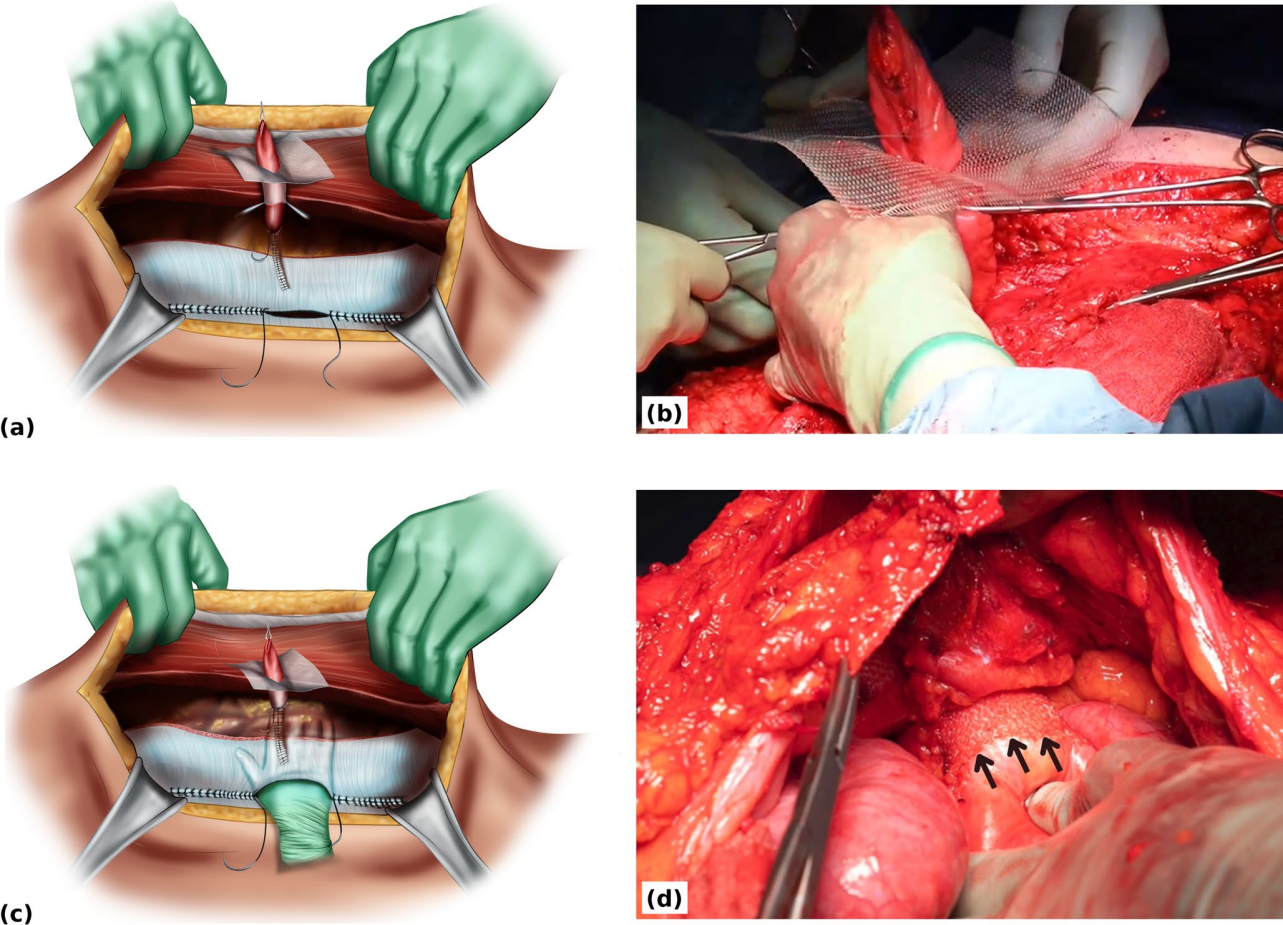
Retromuscular placement of the 3D funnel mesh. (**a**) The stomal conduit is delivered through the funnel of the 3D funnel mesh, the funnel is oriented downward (**b**) Intra-operative view showing positioning of the 3D funnel mesh with the stomal conduit passing through the funnel, which is directed downward toward the peritoneal cavity (**c**) The portion of stomal conduit within the mesh funnel is guided back through the 2 cm lateral opening in the posterior layer into the peritoneal cavity via the midline gap (**d**) Intra-operative view showing that only the mesh funnel sits in the peritoneal cavity. Black arrows indicate the free edge of the funnel intraperitoneally. The flat portion of the 3D mesh remains in the retromuscular space lying on top of the posterior layer

**Fig. 4:**
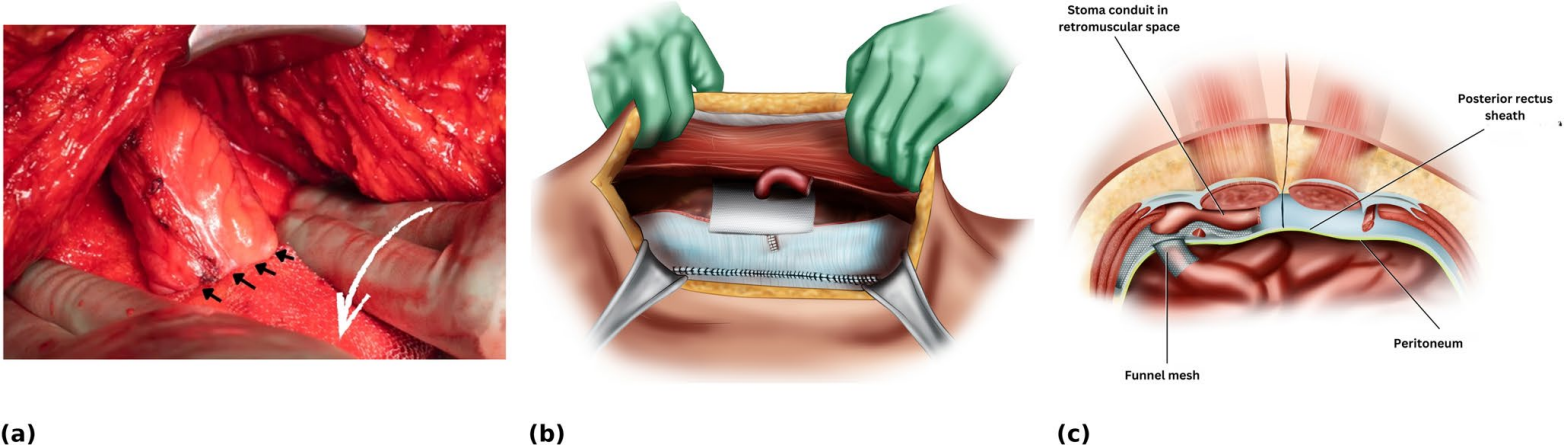
Final retromuscular funnel-mesh configuration. (**a**) Intra-operative view of the 3D funnel mesh within the retromuscular space. The white arrow indicates the flat portion of the 3D funnel mesh lying on top of the posterior layer. Black arrows highlight the funnel aperture, oriented inward toward the peritoneal cavity with the stomal conduit passing snugly through it (**b**) The 15 × 15 cm mesh lies in the retromuscular space against the posterior layer, with midline closure of the posterior rectus sheath completed (**c**) Cross-sectional illustration showing the 4 cm funnel positioned intraperitoneally, the flat portion of the mesh on the posterior layer, and the stomal conduit traversing the retromuscular space

The stomal conduit is delivered through the funnel of the 3D mesh, with the funnel oriented downwards (Fig. 3a, b). The portion of stomal conduit within the mesh funnel is guided back through the 2 cm lateral opening in the posterior layer into the peritoneal cavity using the remaining 10 cm midline gap (Fig. 3c, d). The flat portion of the 3D mesh, measuring 15 × 15 cm, lies in the retromuscular space flat against the posterior layer (Fig. 4a, b).

The opening around the meshed stomal conduit is further suture closed to sit snugly around the meshed conduit. The remaining 10 cm gap in the posterior midline is suture closed (Fig. 4b, c).

**Fig. 5:**
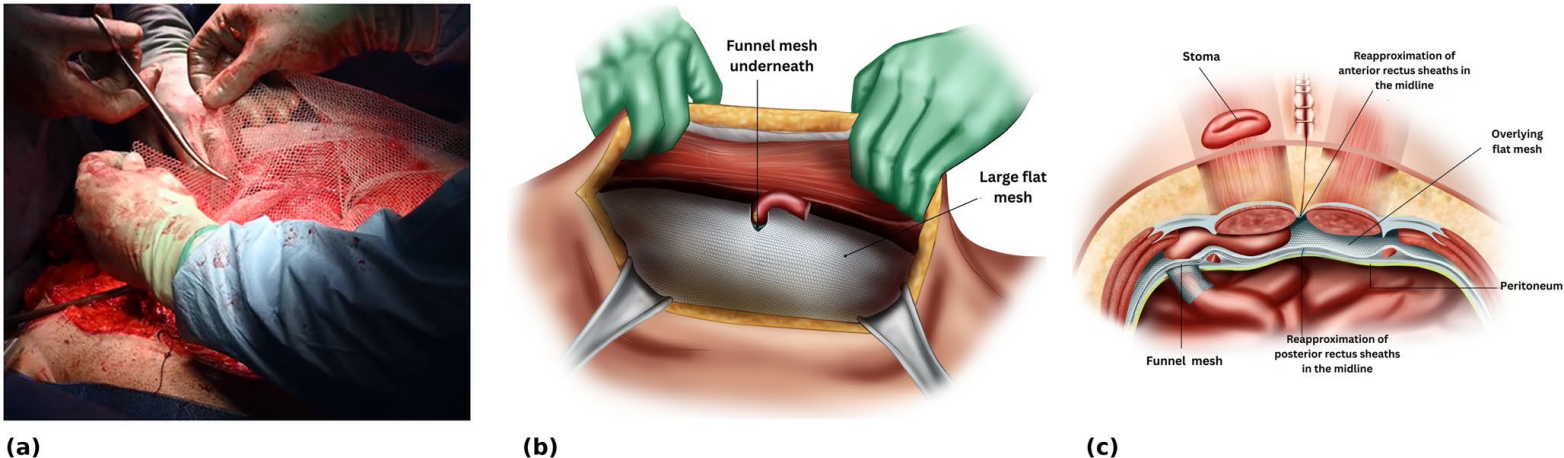
Additional mesh reinforcement and final stoma positioning. (**a**) Intra-operative view of second-mesh fashioning and placement. A slit is fashioned to accommodate and fit snugly around the stoma conduit (**b**) The second larger flat mesh is placed to cover the remaining retromuscular space and overlap the 3D funnel mesh within the retromuscular plane (**c**) Cross-sectional view showing both the funnel mesh and the overlying flat mesh, with the stomal conduit passing through the original trephine in the rectus muscle and anterior sheath

A second larger flat mesh is placed to cover the remaining retromuscular space. This overlaps the funnel mesh, with a slit fashioned so as to wrap around the stoma conduit (Fig. 5a, b).

The stomal conduit is then passed through the original trephine in the rectus muscle and anterior sheath (Fig. 5c).

Two closed suction drains are placed over the meshes in the retromuscular space.

Stage 5 – Anterior Layer and Skin Closure; Stoma Refashioning.

The anterior rectus sheath is closed in the midline. The lateral edge of the trephine in the anterior rectus sheath is checked for impingement on the stomal conduit and released if necessary. The medial edge of the trephine is suture closed up to the conduit, ensuring a snug fit.

The stomal conduit is either brought through the original skin trephine, or if an abdominoplasty was planned, it is completed at this stage. A new stoma site is positioned on the skin at a site pre-determined by the patient and the stoma nurses. The skin opening is made circular, matching the diameter of the stomal conduit but no larger than 2–3 cm [9].

Closed suction drains are placed in the subcutaneous space before skin closure.

After skin closure, the stoma is refashioned. It is spouted 2–2.5 cm for an ileostomy and 1–1.5 cm for a colostomy.

### Results

A total of 15 patients underwent open retromuscular repair with funnel mesh reinforcement. The median age was 59 years (range, 48–71), and 10 patients (66.7%) were female. The median ASA score was 2 (ASA II: 73.3%; ASA III: 26.7%). The median body mass index (BMI) was 33.8 kg/m² (range, 28.4–36.2). (Table 1).

**Table 1:** Patient demographics and hernia characteristics

Variable	Value
**Total patients**	15
**Median age (years)**	59 (48–71)
**Sex**	
• Female	10 (66.7%)
• Male	5 (33.3%)
**BMI (kg/m²)**	33.8 (28.4–36.2)
**ASA (median)**	2
• ASA II	11 (73.3%)
• ASA III	4 (26.7%)
**Stoma type**	
• Colostomy	9 (60.0%)
• Ileostomy	6 (40.0%)
**Recurrent hernia**	5 (33.3%)
**Concomitant incisional hernia**	13 (86.7%)
**Combined defect width (cm)**,** median [IQR]**	11.9 [10.4–12.2]
**Parastomal defect length (cm), median [IQR]**	5.4 [4.8–6.0]
**Parastomal defect width (cm)**,** median [IQR]**	4.4 [3.4–5.2]
**Midline defect length (cm)**,** median [IQR]**	9.2 [6.7–11.8]
**Midline defect width (cm)**,** median [IQR]**	5.1 [4.1–6.1]
Botox* administered	14 (93.3%)

Values are presented as *n* (%) or median (range)/median [IQR] unless otherwise stated

*ASA *American Society of Anesthesiologists,* BMI *body mass index. *Botulinum toxin A

Stoma type was colostomy in 9 patients (60.0%) and ileostomy in 6 patients (40.0%). Five patients (33.3%) presented with a recurrent parastomal hernia, including two who had undergone more than one previous repair. Thirteen patients (86.7%) had a concomitant incisional hernia: 12 midline and one at a previous right iliac fossa ileostomy site. In this latter case, the stoma had been relocated from right to left, with subsequent hernia formation at both sites.

Two patients with para-ileostomy hernia underwent simultaneous procedures: one completion proctectomy and one subtotal colectomy.

Preoperative botulinum toxin A was administered in 14 patients (93.3%).

Abdominoplasty was performed in 13 patients (86.7%), most commonly via a vertical approach, with two patients undergoing a fleur-de-lis procedure (Table 2). 

**Table 2:** Perioperative details and outcomes

Variable	Value
**TAR**	15 (100%)
• Bilateral	8 (53.3%)
• Unilateral	7 (46.7%)
**Mesh used**	
• Funnel mesh (DynaMesh-IPST^®^)	15 (100%)
• Synthetic (Dynamesh- CICAT^®^)	11 (73.3%)
• Biosynthetic (BioA^®^)	4 (26.7%)
**Abdominoplasty**	13 (86.7%)
• Vertical/midline ellipse	11 (73.3%)
• Fleur-de-lis	2 (13.3%)
**Median hospital stay (days)**	7 (range 6–8)
**Median follow-up (months)**	15(range 4–32)
**Recurrence**	0 (0%)
**Complications**	
• Wound infections	2 (13.3%)
• Collection/Abscess	1 (6.7%)
• Infected seroma	1 (6.7%)
• Stoma prolapse	1 (6.7%)
• Stomal retraction	1 (6.7%)

Values are presented as *n* (%) or median [range] unless otherwise stated. *TAR* transversus abdominis release

The median parastomal fascial defect length and width were 5.4 cm (IQR 4.8–6.0.8.0) and 4.4 cm (IQR 3.4–5.2), respectively. The corresponding median midline defect length and width were 9.2 cm (IQR 6.7–11.8) and 5.1 cm (IQR 4.1–6.1). The median combined transverse defect width (parastomal and midline, where present) was 11.9 cm (IQR 10.4–12.2).

TAR was performed bilaterally in 8 patients (53.3%) and ipsilaterally in 7 patients (46.7%). All patients received a DynaMesh-IPST^®^ funnel mesh. In all cases, an additional synthetic flat mesh was used to fully cover the retromuscular space: permanent synthetic (DynaMesh-CICAT^®^) in 11 patients (73.3%) and slowly resorbable (Gore Bio A^®^ tissue reinforcement) in 4 patients (26.7%).

No intra-operative complications occurred, and there were no 30-day mortalities.

The median length of stay was 7 days (range, 6–8). Follow-up was conducted by clinical assessment in all patients. During the follow-up period, seven patients (46.7%) had an abdominal CT scan, either to evaluate persistent symptoms or for other non–hernia-related indications. At a median follow-up of 15 months (range, 4–32), no parastomal hernia recurrences, stomal stenoses, or obstructions were observed. Postoperative complications included superficial wound infection managed with antibiotics (*n* = 2), a flank abdominal wall collection requiring radiological drainage (*n* = 1), an infected seroma overlying the mesh successfully managed by open drainage and negative pressure wound therapy (NPWT) (*n* = 1), stoma conduit prolapse into the subcutaneous space without fascial recurrence (*n* = 1) and stoma conduit retraction (*n* = 1) (Table 2). Both these latter 2 patients required re-operation for refashioning of the stomal conduit, with an uneventful recovery.

## Discussion

When repairing parastomal hernias the spectrum of patients’ symptoms should be considered in addition to the traditional surgeon focused measures of success, such as hernia recurrence and surgical site infection (SSI). Patient symptoms have been categorised into themes of physical sensation including pain, stoma bag difficulties leading to leakages and skin irritation, and cosmesis affecting body image, self-consciousness and low confidence. Unless these are addressed then irrespective of hernia recurrence and SSI, the patient may still deem the operation to have been a failure [10, 11].

To improve the range of patients’ symptoms, the following components must be considered: the fascial defect and the herniated components; the additional features making up the peristomal ‘bulge’, which include excess skin, subcutaneous fat, and the hernia sac, all of which can interfere with stoma bag application and adherence; and the stomal conduit itself.

We believe there are three essential principles for effective elective parastomal hernia repair that encompass the above components:

1. Adherence to the fundamental principles of non-parastomal incisional hernia repair, which include reduction of the hernia contents, excision of the hernia sac (unless needed for defect closure), and mesh-reinforced fascial closure.
2. Balanced mesh reinforced fascial closure of the stomal trephine that avoids both excessive narrowing (risking obstruction or ischaemia) and leaving the trephine too wide (predisposing to recurrence).
3. Creation of a well-spouted stoma on a flat surface, optimising stoma bag application, adherence and skin protection [9].

Expert opinion suggests that the intraperitoneal and retromuscular planes are the preferred locations for mesh placement [1].

The intraperitoneal 3D funnel mesh achieves low recurrence and SSO rates compared with other intraperitoneal mesh configurations and has been applied across all EHS PSH classifications [3, 12]. However, there is increasing consensus that large parastomal hernias (EHS PSH type III and IV) or smaller parastomal hernias with large concomitant midline hernias (EHS PSH type II with EHS midline hernia classification W2 or W3) may be more appropriately treated via retromuscular reconstruction, which allows a more anatomical repair and avoids intraperitoneal mesh placement with its potential visceral complications.

To date, three retromuscular techniques have been described: keyhole mesh, the Sugarbaker configuration, and Stapled Transabdominal Ostomy Reinforcement with Retromuscular Mesh (STORRM) [4, 5, 13].

In a recent randomised controlled trial, 2-year recurrence rates were 24% for keyhole and 17% for Sugarbaker mesh configurations, with no statistically significant differences in reoperation for recurrence, non-hernia related intra-abdominal pathology, or mesh-related complications. Importantly, both groups reported improved quality of life without decision regret [6].

The STORRM technique is technically demanding and more costly, with no demonstrated advantage over the other two retromuscular mesh repair approaches [13].

The retromuscular 3D funnel mesh technique described combines the strengths of the retromuscular approaches and the intraperitoneal 3D funnel mesh repair, whilst also addressing:

- recurrence mechanisms recognised with the retromuscular keyhole and Sugarbaker configurations.
- potential visceral complications associated with intraperitoneal mesh.
- the enlarged anterior rectus sheath trephine.
- the peristomal “bulge” of redundant skin, subcutaneous fat, and hernia sac that can compromise stoma appliance application and adherence.

Some may argue that lateralising the stomal opening in the posterior layer is unnecessary, and that the funnel mesh could be placed directly in the retromuscular space. However, we believe that offsetting the posterior and anterior openings provides a mechanical advantage. This principle reflects the Sugarbaker repair and parallels the offset configuration of the superficial and deep rings of the inguinal canal.

To our knowledge, this is the first report of retromuscular placement of a 3D funnel mesh for parastomal hernia repair. By combining the advantages of the intraperitoneal 3D funnel mesh and the retromuscular Sugarbaker repair, we propose the term ‘Sugar Funnel repair’.

This technique was performed in an open manner, but it could potentially be adapted to minimally invasive surgery, much as the retromuscular Sugarbaker technique was initially described open before being reproduced successfully robotically [14, 15].

The choice of the second flat mesh to fully cover the retromuscular space was based on both surgeon and patient preference. The rationale for the mesh selection described in this article have been previously published [16]. The use of different mesh types may represent a limitation in this small cohort of patients. Another limitation is that, although post-operative clinical assessment was undertaken in every case and a CT scan when clinically indicated, a standardized imaging protocol to detect radiological recurrence was not performed.

Early outcomes in our cohort are encouraging. At a median follow-up of 15 months, no true recurrences or stomal conduit obstructions were observed. Complications were limited to wound morbidity and stomal conduit prolapse or retraction. These results compare favourably with contemporary registry benchmarks [2]. Importantly, functional outcomes such as stoma appliance fit, the quality-of-life impact of contour correction, and skin care were considered in our approach, although these were not formally assessed.

However, this technique is complex and requires expertise in TAR, parastomal hernia surgery in general, and abdominoplasty, making it best suited to specialised abdominal wall reconstruction centres with multidisciplinary support.

In conclusion, the retromuscular 3D funnel mesh technique offers a robust repair with potential advantages over other recognized repair options, for primary and recurrent parastomal hernias and especially those with concomitant incisional hernias. Registry based data, larger series with longer follow-up, and ultimately comparative studies primarily focused on patient reported outcome measures are needed to confirm these early findings. 

## Data Availability

The datasets generated and/or analysed during the current study are available from the corresponding author on reasonable request.
